# Paradoxical Skin Reactions to Biologics in Patients With Rheumatologic Disorders

**DOI:** 10.3389/fphar.2019.00282

**Published:** 2019-03-26

**Authors:** Simone Garcovich, Clara De Simone, Giovanni Genovese, Emilio Berti, Massimo Cugno, Angelo Valerio Marzano

**Affiliations:** ^1^Institute of Dermatology, Fondazione Policlinico Universitario A. Gemelli IRCCS, Università Cattolica del Sacro Cuore, Rome, Italy; ^2^UOC Dermatologia, Fondazione IRCCS Ca’ Granda Ospedale Maggiore Policlinico, Milan, Italy; ^3^Dipartimento di Fisiopatologia Medico-Chirurgica e dei Trapianti, Università degli Studi di Milano, Milan, Italy; ^4^Medicina Interna, Fondazione IRCCS Ca’ Granda Ospedale Maggiore Policlinico, Milan, Italy

**Keywords:** paradoxical skin reactions, biologics, rheumatological disorders, psoriasis, TNFα-inhibitors

## Abstract

Targeted immune-modulating treatment with biological agents has revolutionized the management of immune-mediated inflammatory diseases, including rheumatologic conditions. The efficacy and tolerability of biological agents, from the initial tumour necrosis factor (TNF)-α inhibitors to the new anti-cytokine monoclonal antibodies, have dramatically changed the natural history of debilitating conditions such as rheumatoid arthritis and seronegative spondyloarthropathies. The widening use of biologics across several rheumatologic diseases has been associated with a new class of adverse events, the so-called paradoxical reactions. These events are inflammatory immune-mediated tissue reactions, developing paradoxically during treatment of rheumatologic conditions with targeted biologics that are commonly used for treating the idiopathic counterparts of these drug-induced reactions. The skin is frequently involved, and, even if considered rare to uncommon, these cutaneous manifestations are an important cause of biologic agent discontinuation. TNF-α antagonist-induced psoriasis, which can manifest *de novo* or as exacerbation of a pre-existing form, is the prototypic and most frequent paradoxical skin reaction to biologics while other reactions, such as eczematous and lichenoid eruptions, hidradenitis suppurativa, pyoderma gangrenosum, Sweet’s syndrome and granulomatous skin diseases, occur much more rarely. Management of these reactions consists of topical or systemic skin-directed therapies, depending on the severity and extension of the cutaneous picture, and it is generally associated with switching over to other disease-modifying regimens for treating the underlying rheumatologic condition. Here, we review in detail the current concepts and controversies on classification, pathogenesis and clinical management of this new class of cutaneous adverse events induced by biologics in rheumatologic patients.

## Introduction

Targeted biological agents have dramatically changed the treatment landscape of immune-mediated inflammatory diseases (IMIDs) with rheumatological conditions being at the front of this revolution. The efficacy and tolerability of targeted biological agents have determined a paradigm shift in the treatment of several rheumatologic conditions, modifying the natural history of progressive, invalidating disease, such as rheumatoid arthritis (RA) and seronegative spondyloarthropathies (SpA). While biological agents (BA) have a superior safety and tolerability profile compared to conventional disease-modifying anti-rheumatic drugs (DMARDs), they may cause different cutaneous adverse events, either of infectious, inflammatory or neoplastic origin (Hernandez et al., 2013). Furthermore, targeted treatment with BA is increasingly associated with a new class of adverse events, the so-called paradoxical immune-mediated inflammatory reactions. Paradoxical reactions (PR) are defined by the development of inflammatory immune-mediated tissue manifestations in IMID patients treated with targeted biological agents. The skin is frequently involved by this paradoxical inflammation, as in the case of plaque psoriasis developing in a rheumatological patient during treatment with TNF-α inhibitors (TNFi) (Viguier et al., 2009).

Cutaneous PR have been described as a class-effect of targeted BA, especially TNFi, first in rheumatologic patients and subsequently across all other indications, such as psoriasis and inflammatory bowel-disease (IBD). Reports of different, organ-specific PR are constantly increasing, as long-term use of new anti-interleukin (anti-IL-6, -IL-17,-IL-12/23) and first-to-second generation TNFi biosimilars is growing (Toussirot and Aubin, 2016). Furthermore, cutaneous PR represent an intriguing immunological and clinical dilemma, whose unraveling may improve our knowledge of the pathogenesis of chronic inflammatory diseases. These puzzling cutaneous eruptions may also represent a new type of adverse drug reactions in the era of precision medicine, resulting from the interaction between targeted manipulation of cytokine-molecular networks by BA and patient’s genetic predisposition (Cabaleiro et al., 2016). We review the clinical spectrum of paradoxical cutaneous inflammation induced by targeted BA in patients with rheumatological disorders, discussing the current controversies on classification, pathogenesis and clinical management.

### Cutaneous Paradoxical Reactions: Definition and Scope of the Problem

Psoriasis, and its clinical variants, represents the prototypical cutaneous PR, as this was the first paradoxical reaction pattern described in rheumatologic patients treated with the first-generation BA, namely the TNFi. Therefore, most clinical and experimental studies on PR have focused on paradoxical psoriasis, providing the conceptual framework for the other cutaneous PR. The literature on epidemiology of PRs in patients treated with BA is scarce, as most of the clinical evidence derives from retrospective studies, case series and reports. The estimated prevalence of cutaneous PR ranges from 0.6 to 5.3% in patients treated with TNFi (Sfikakis et al., 2005; Fouache et al., 2009; Ko et al., 2009; Famenini and Wu, 2013; Bae et al., 2018). In a registry-based observational study, the incidence of paradoxical psoriasis in RA patients treated with TNFi has been estimated in 1.04 per 1000 persons/years. Patients treated with TNFi presented an incidence rate ratio (IRR) of 2.0–5.94 for the onset of paradoxical psoriasis compared to patients treated with conventional DMARDs (Hernandez et al., 2013). In RA patients, the incidence of paradoxical psoriasis has been estimated in one new case for every 550 patients treated with adalimumab per year (Harrison et al., 2009). In the context of adverse drug reaction, cutaneous PRs could be classified as uncommon-to rare events. Typically, cutaneous PRs, such as paradoxical psoriasis, can be induced *de novo* in rheumatologic patients without a history of cutaneous inflammatory disease during treatment with a BA. On the other hand, cutaneous PR can be an exacerbation, with or without a change in clinical morphology, of a pre-existing cutaneous inflammatory disease in a genetically- predisposed patient. This is the case of paradoxical palmoplantar pustular psoriasis developing during TNFi in a RA patient with a history of plaque psoriasis. Key features supporting the causal relationship between a skin PR and therapy with a BA are: (a) the temporal association and (b) clinical outcome of the PR after BA withdrawal. Cutaneous PR can occur at any time during treatment with a BA, but more than 60% of cases of paradoxical psoriasis has been reported to develop within the first year of treatment (Brown et al., 2017). As observed in cutaneous drug reactions, the cessation of the triggering “culprit” BA determines clinical resolution or improvement of the skin PR. Re-treatment, or drug re-challenge, with the same BA or related BA-class has been associated with the relapse of paradoxical skin inflammation.

## Classification of Cutaneous Paradoxical Reactions

**FIGURE 1:**
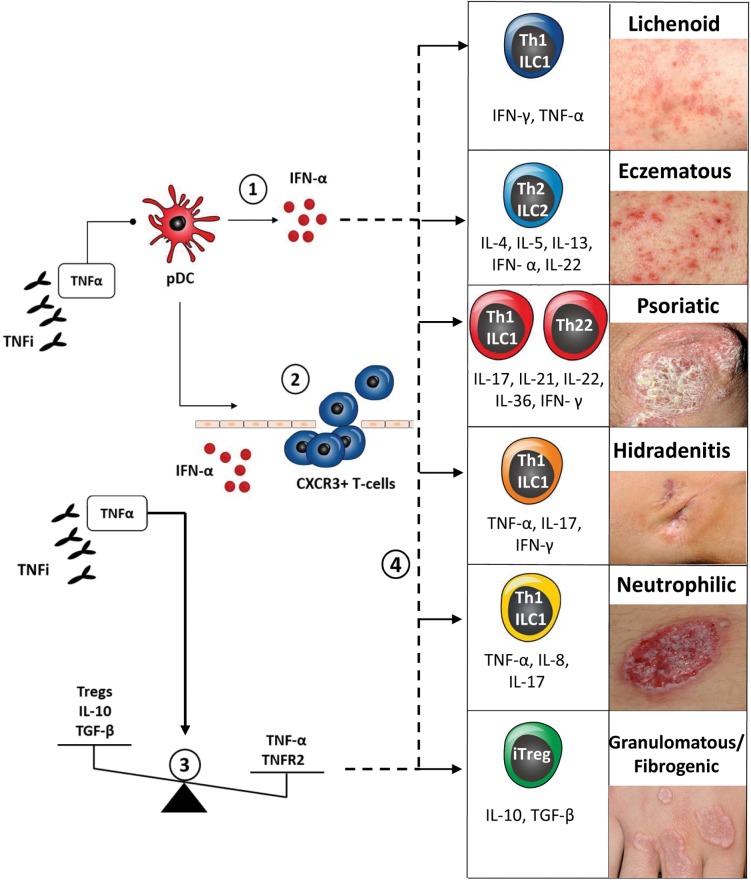
Classification and pathogenesis of cutaneous paradoxical reactions to biologic agents (^∗^) and their related key immune-response/cytokine patterns. (1) TNF-α/type-1 IFN cytokine imbalance: pharmacological blockade of TNF-α by TNF-inhibitors (TNFi) determines uncontrolled activation of plasmacytoid dendritic cells (pDCs), with sustained production of IFN-α. IFN-α drives paradoxical skin inflammation via downstream effectors cytokines, thus eliciting key cutaneous immune response patterns → clinical correlation: paradoxical psoriasis and psoriasiform eruptions. (2) IFN-α induced spatial shift of activated innate immune cells and chemokine (C-X-C motif) receptor 3 (CXCR3) positive T-cells from extra-cutaneous tissue compartments to the skin → clinical correlation: *de novo* induction of psoriasiform eruptions and hidradenitis suppurativa. (3) TNF-α/IL-10 cytokine imbalance and Tregs and TNFR2 imbalance → clinical correlation: paradoxical cutaneous sarcoidosis and granulomatous disease. (4) Shift in cutaneous immune response pattern → clinical correlation: *de novo* induction of lichenoid, eczematous PR or change in psoriatic morphology (plaque to pustular). (^∗^) Based on expert opinion and on current available clinical and experimental evidence; cutaneous immune response patterns adapted from Eyerich and Eyerich (2018).

In rheumatologic patients, cutaneous PRs induced by BA can present with different clinical aspects and extent, involving the skin, its appendages and transitional epithelial surfaces. Cutaneous PRs, as defined previously, encompass a variety of inflammatory manifestations/conditions, which can be both treated and triggered with the same cytokine-targeted BA. Cutaneous PRs reported in rheumatologic patients are summarized in Figure 1 and include psoriasis and its spectrum of clinical phenotypes (plaque, pustular, generalized, palmoplantar, scalp, guttate, and inverse), hidradenitis suppurativa (HS), neutrophilic dermatosis (the prototypical forms pyoderma gangrenosum and Sweet’s syndrome) and granulomatous skin disease (granuloma annulare, interstitial granulomatous dermatitis, necrobiosis lipoidica, and sarcoidosis). Other BA-inducible cutaneous inflammatory conditions, such as atopic dermatitis, cutaneous vasculitis, drug-induced lupus erythematosus and other allergic and hypersensitivity reactions are not strictly considered “paradoxical” because their idiopathic counterparts are not generally treated with these agents.

Classification of inflammatory skin disease is traditionally based on clinical morphology of primary and secondary skin lesions in combination with a histological description of epidermal-dermal tissue involvement and underlying pathomechanisms (Dainichi et al., 2014). Adverse cutaneous drug reactions also share similar classification systems, with a combination of clinical descriptors of lesion morphology (psoriasiform, bullous etc.) histological pattern (spongiotic, lichenoid/interface dermatitis, etc.) and underlying predominant immunologic/hypersensitivity mechanism (type I-IV reaction) (Isabwe et al., 2017). A recent trend in the classification of inflammatory skin disease is to integrate clinicopathological data with molecular-immunologic information, such as predominant disease cell-subset, cytokine expression patterns and molecular biomarkers (Inkeles et al., 2015; Garzorz-Stark and Lauffer, 2017) Recently, Eyerich and Eyerich (2018) summarized the cutaneous immune-response patterns (Th1-, Th2-, Th17/Th22- and Treg-cells and related cytokines) associated with specific cutaneous-tissue response patterns (lichenoid, eczematous/blistering, psoriatic, fibrogenic/granulomatous), providing a molecular-pathophysiological approach to the traditional, complex dermatological nosology. This conceptual classification can be used for the description of cutaneous PR, along with its prevalently associated clinical and immune-response patterns, according to currently published data (refer to Figure 1). According to its initial descriptions, cutaneous PRs can be considered almost identical to its corresponding, “classic” inflammatory skin disease in terms of clinical, histological and immunological presentation. In the following sections we will discuss some limitations of this concept, based on recent clinical and experimental studies.

In BA-treated patients, the clinical picture of cutaneous PR may vary from typical inflammatory skin lesions - clinically and histological identical to its correspondent primary, non-BA induced skin disease – to atypical inflammatory skin manifestations, with “overlapping” clinical and histological features. For example, paradoxical TNFi-induced psoriasis may present with a wide clinical spectrum, with typical erythematous-squamous or pustular lesions, clinically indistinguishable from conventional plaque or pustular psoriasis, to atypical papulo-squamous eruptions with “psoriasiform,” “eczematous” or “lichenoid” lesion morphology (Succaria and Bhawan, 2017). Correlation with histological aspects of lesional skin is crucial for diagnosis of the PR type and differentiation with other cutaneous adverse events. Moreover, in most published series the spectrum of histological changes of a “psoriasiform” paradoxical eruption is quite variable, ranging from typical psoriatic pattern to lichenoid/interface dermatitis, pustular folliculitis and eosinophil-rich perivascular dermatitis pattern. Psoriatic alopecia has been recently described as a distinct clinical phenotype of anti-TNF-α-induced, paradoxical psoriasis, presenting with patchy, non-cicatricial alopecia due to marked inflammatory involvement of the scalp skin and hair follicles (Figure 1; Osório et al., 2012; George et al., 2015). Histologically, features of both conventional scalp psoriasis and alopecia areata have been observed (Doyle et al., 2011).

Lichen planus (LP)-like or lichenoid skin eruptions are characterized by an interface dermatitis histological pattern and prominent Th1/ILC1 (type 1 innate lymphoid cell)-IFN (interferon)-γ-biased immune-response. This skin-directed cytotoxic reaction can be triggered by microbial antigens, xenobiotics and drugs. Paradoxical, lichenoid eruptions have been reported in RA and psoriatic arthritis (PsA) patients during treatment with TNFi, variably involving the skin, oral/genital mucosa, nails and hair-follicles (Vergara et al., 2002; Asarch et al., 2009; Garcovich et al., 2010).

Hidradenitis suppurativa is a chronic, inflammatory disease of the follicular epithelium, presenting with suppurative lesions (nodules, abscesses, pustules, fistulas, sinus-tracts, and hypertrophic scars) affecting the skin folds and anogenital area. The cutaneous immune response pattern of primary HS is characterized by Th17/ILC3 lymphocyte subset, with strong IL-1β, TNF-α, IL-17 cytokine-signature, and peripheral recruitment of IL-17-producing neutrophils and Th17-cells. Paradoxical HS has been recently reported in patients with RA or spondyloarthropathies (PsA, AS, SAPHO) during long-term treatment (mean 25 months) with TNFi and other BA (tocilizumab, rituximab) (Delobeau et al., 2016; Faivre et al., 2016). Most patients presented known risk factors for HS (smoking, overweight), but the relapse of paradoxical HS after re-treatment with involved BA supports causality.

Pustular psoriasis and neutrophilic dermatoses (pyoderma gangrenosum, Sweet syndrome) present a sterile pustule as hallmark cutaneous lesion of neutrophilic inflammation. Both “neutrophilic” inflammatory conditions share common downstream inflammatory mediators, such as TNF-α, IL-8, IL-17, IL12/23 and IL-36, which promote activation and migration of neutrophils in the skin. Pyoderma gangrenosum, the prototypical form of hypodermal neutrophilic, can be both treated and paradoxically triggered by almost all the TNFi (etanercept, adalimumab, infliximab, golimumab) (Vandevyvere et al., 2007; Brunasso et al., 2010; Kowalzick et al., 2013; Marzano et al., 2018; Skalkou et al., 2018).

Granulomatous skin conditions are a heterogenous group of chronic inflammatory diseases, which include also reactive or drug-induced processes. Reactive granulomatous skin eruptions have a wide spectrum of clinical morphologies with several clinical entities, such as interstitial granulomatous dermatitis (IGD) and palisaded neutrophilic and granulomatous dermatitis (PNGD). These reactive conditions can be triggered by systemic inflammatory conditions, such as connective tissue disease and the rheumatic disease, and by several drugs, such as TNFi (Deng et al., 2006). Localized and generalized forms of granuloma annulare have been reported in rheumatologic patients during treatment with TNFi (Voulgari et al., 2008). Histological evidence of dermal non-caseating granulomas is a hallmark of cutaneous sarcoidosis, which can present several clinical-morphological variants (Amber et al., 2015; Rosenbach and English, 2015). Paradoxical development of sarcoidosis-like skin lesions has been reported in rheumatologic patients treated with TNFi, especially with etanercept (Dhaille et al., 2010; Massara et al., 2010; Robicheaux Clementine et al., 2010; Lamrock and Brown, 2012; Decock et al., 2017). In sum, cutaneous PR presents a wide spectrum of clinical-histological reaction patterns.

## Pathogenesis of Cutaneous Paradoxical Inflammation

Since the initial descriptions, cutaneous inflammatory disease presenting *de novo* in rheumatologic patients during treatment with potent, cytokine-targeted BA represented a clinical and immunological conundrum. Considering the molecular taxonomy of inflammatory skin disease, cutaneous PR can be explained by the complex interplay between host-specific factors (genetic predisposition) and BA-induced specific shifts in cytokine and cellular immune-response patterns ( Palucka et al., 2005; Grine et al., 2015; Verwoerd et al., 2016). According to current experimental data, cutaneous paradoxical inflammation may result from different putative immune-pathogenetic mechanisms (summarized in Figure 1), leading to different types of clinical reactions. BA- induced immune-pathogenesis of cutaneous PRs include one or more of the following mechanisms as *primum movens*: (a) a cytokine imbalance; (b) a shift in cutaneous immune response pattern; (c) a spatial shift of activated innate or adaptive immune cells to the skin; (d) imbalance or dysfunction of regulatory T-cells.

In the case of paradoxical psoriasis, a TNFi-induced cytokine imbalance between TNF-α and type 1-Interferons (IFN-α) has been reported as key-pathogenetic factor (Marzano et al., 2014). Lesional skin of psoriasiform PR displayed an increased tissue-expression of MxA (myxovirus-resistance protein A), i.e., type-1 interferon activity (de Gannes et al., 2007). Notably, systemic treatment with recombinant IFN-α and topical application of IFN-α inducers (imiquimod) are both able to elicit psoriatic skin lesions in clinical and experimental models (Gilliet et al., 2004). Continuous therapeutic TNF-α blockade thus cause a specific, cutaneous cytokine imbalance, favoring development of inflammatory plasmacytoid dendritic cells (pDCs) and increased type-1 IFN production (Conrad et al., 2018). Increased IFN-α-activity in turn determines abnormal trafficking and homing of pDCs and innate immune cells to the skin, in an inflammatory loop. Furthermore, in RA patients, treatment with TNFi has been associated with increased expression of the chemokine receptor CXCR3 on peripheral T-cells, potentially favoring trafficking of activated T-cells to the skin (Aeberli et al., 2005). This dysregulated, paradoxical innate immune response may then translate clinically to paradoxical psoriasis in genetically susceptible subjects.

Recent experimental studies raised several controversies on the true nature of cutaneous PR, differentiating its immune pathogenesis from their “classical,” non-paradoxical counterparts. Stoffel et al. (2018) reported both psoriasiform and eczematous PR to have a distinct lesional immune response pattern, with a common strong Th1- and type-1 IFN pattern, differing, respectively, from “classic” psoriasis and eczema controls. In the same study, psoriasiform PR presented an increased expression of type 1 IFN (IFN-α, IFN-γ) and pro-inflammatory cytokines (IL-36, IL-19, IL-20), whereas eczematous PR were associated with up-regulated Th-2 cytokines (IL-13, IL-5) and IL-22 expression. In psoriasiform PRs, the involvement of IL-1-(IL-36) and IL-17 (IL-17A) cytokine families, sustaining a pro-inflammatory loop mechanism, has been also reported in patients with IBD and psoriasis (Tillack et al., 2014; Deubelbeiss et al., 2018). Finally, the group of Gilliet et al. (2004) designed an experimental murine model for paradoxical skin inflammation to support the differences between paradoxical psoriasis and “classic” psoriasis. In this model, induction of the “paradoxical” psoriasiform phenotype is mainly driven by type 1 IFN expression and cutaneous infiltration of pDCs due to temporal TNF-α/IFN imbalance. The resulting paradoxical psoriasiform skin inflammation is mostly independent from T-cells, i.e., from adaptive immune responses, which is in contrast with “classical” psoriasis (Conrad et al., 2018). BA-induced shifts of cutaneous immune response patterns may then interact with host-specific genetic risk variants for inflammatory cutaneous disease, promoting the development of PRs. Indeed, preliminary studies support the role of specific IL-23 receptor (IL-23R) gene polymorphisms to be linked to anti-TNF-α-induced paradoxical psoriasis (Sherlock et al., 2013).

## Clinical Implications of Paradoxical Reactions to Biological Agents

Cutaneous PR occurring during treatment with BA represents a clinical challenge in terms of differential diagnosis and management. Clinical management should be aimed at treating the cutaneous signs (eruption) and symptoms (pain, pruritus etc.) while maintaining control of the underlying rheumatologic condition. As discussed previously, the modification of the anti-rheumatic treatment regimen, i.e., a treatment suspension/withdrawal or therapeutic switch, is associated in most cases with an improvement or resolution of the cutaneous PR. Therefore, when approaching the rheumatologic patient with a PR, the clinician should take into account several factors, including: (a) the extent and severity of skin involved by the PR, (b) the severity and activity of the background rheumatologic condition, (c) the patient’s quality of life and comorbidities, (d) the possible loss of efficacy of the culprit BA in the case of cessation/retreatment, (e) the availability of alternative treatment options for the rheumatologic condition. Dose-reduction and discontinuation strategies of BA in rheumatologic patients should be evaluated on a per-case basis by the treating rheumatologist, as there are no definitive guidelines for the management of cutaneous PR (van Herwaarden et al., 2014). In the case of anti-TNF-α induced psoriasis, a practical treatment algorithm has been initially proposed by Collamer et al. (2008) and this can be adapted to other cases of cutaneous PRs as well.

In a BA-treated rheumatologic patient developing inflammatory skin lesion a high-grade of suspicion for PR should be taken. Interdisciplinary care should necessary include evaluation by a dermatologist and lesional skin biopsy, to aid clinical-histological correlations and differential diagnosis with other cutaneous adverse events. The severity of cutaneous PR can be graded with simple assessments, such as the extent of body surface area (BSA) involved, symptom-based scores (pruritus/pain intensity scores) and patient-reported outcomes (dermatology life quality index [DLQI]). The addition of a skin-directed therapy (topical or systemic) is a reasonable initial strategy to manage the cutaneous PR and to maintain the patient on treatment with the BA. Topical skin-directed therapies (topical steroids, keratolytic agents, immunomodulators, vitamin D analogs) are a viable option for PR with mild (BSA < 5%) to moderate (BSA 5–10%) skin involvement, such as localized plaque psoriasis, lichenoid reactions or granulomatous lesions. In the case of PR with moderate-to severe skin involvement (>10% BSA), progressive course and/or high symptom-burden (QoL impairment) treatment can be escalated with the addition of UV-phototherapy or traditional systemic agents, such as methotrexate, retinoids (acitretin), cyclosporine and systemic steroids. Combination treatment regimens with systemic, skin-directed agents and ongoing BA (for example TNFi) should be carefully managed in close collaboration by the rheumatologist and the dermatology consultant.

In the case of severe PR with extensive (>10% BSA), unstable disease and/or high disease-burden, treatment with the BA should be discontinued. For example, severe plaque psoriasis, erythrodermic psoriasis, generalized pustular psoriasis or highly pruritic lichenoid or eczematous eruption would necessarily lead to discontinuation of anti-rheumatic treatment with a TNFi. According to published studies, almost 50% of patients will present an improvement or resolution of paradoxical skin lesions, following withdrawal of the BA. Another 45% of patients with anti-TNF-α induced psoriasis may present persistent or recurring cutaneous lesions, despite BA discontinuation. The more severe PR, such as generalized pustular psoriasis or psoriatic alopecia, can run a persistent course, only with partial improvement, after discontinuing the BA (Brown et al., 2017). Re-treatment with the same BA, after cessation of the cutaneous PR, should be evaluated on the basis of concomitant rheumatologic condition and availability of alternative treatment options. There is a substantial risk of recurrence of the cutaneous PR after re-treatment with the same BA, but there is no strong evidence in published studies (Wollina et al., 2008). Therapeutic switch of the PR-triggering BA with another BA of the same class (i.e., alternative TNFi) or of different class can be considered in moderate-to severe cutaneous PR, to control the underlying rheumatologic condition. Therapeutic switch to another BA is also indicated in the severe, paradoxical psoriasis subtypes, as in the case of generalized pustular psoriasis. Switching to a new BA-class, for example from a TNFi to anti-IL6 treatment (tocilizumab), is a common strategy in the management of RA and has been also reported in the case of paradoxical psoriasis (Rueda-Gotor et al., 2012; Shimizu et al., 2015; Cantini et al., 2017).

## Clinical Implications of Paradoxical Reactions to Biological Agents

The unexpected occurrence of paradoxical inflammation during treatment with BA has emerged as a new type of drug-related adverse event, with a complex pathophysiology. The skin is one of the main organs affected by these reactions, presenting with a wide spectrum of clinical and pathological aspects. In rheumatologic patients, cutaneous PRs are frequent and clinically relevant. Adequate clinical management of these reactions is paramount to maintain control of background rheumatologic disease and to improve drug survival of BA. In some cases, therapeutic switch to another class of BA or to new, small-molecule-based disease modifying drugs is warranted to oppose paradoxical inflammation. The understanding of these new types of adverse reactions will hopefully shed light on the complex interactions between host-specific factors (genetic predisposition), immune-mediated comorbidities, immune-regulatory mechanisms and targeted immune-modulation.

## Author Contributions

SG, CS, EB, MC, and AM designed and reviewed the manuscript and contributed in drafting the manuscript. GG contributed in drafting and reviewing the manuscript. All the authors approved the final version of the manuscript.

## Conflict of Interest Statement

The authors declare that the research was conducted in the absence of any commercial or financial relationships that could be construed as a potential conflict of interest.

## Abbreviations

IFN: Interferon
IFN type-1: interferon-type 1
IL: interleukin
IL-10: interleukin 10
ILC: innate lymphoid cell
iTreg: induced regulatory T-cells
NDs: neutrophilic dermatoses
TGF-β: transforming growth factor-beta
Th: T helper cell
TNF-α: tumor necrosis factor-alpha
TNFR2: tumor necrosis factor receptor 2
Tregs: regulatory T-cells
